# Hesperidin Improves Memory Function by Enhancing Neurogenesis in a Mouse Model of Alzheimer’s Disease

**DOI:** 10.3390/nu14153125

**Published:** 2022-07-29

**Authors:** Danbi Lee, Namkwon Kim, Seung Ho Jeon, Min Sung Gee, Yeon-Joo Ju, Min-Ji Jung, Jae Seok Cho, Yeongae Lee, Sangmin Lee, Jong Kil Lee

**Affiliations:** 1 Department of Life and Nanopharmaceutical Sciences, Graduate School, Kyung Hee University, Seoul 02447, Korea; iweks97@naver.com (D.L.); kop03@khu.ac.kr (N.K.); 2 Department of Pharmacy, College of Pharmacy, Kyung Hee University, Seoul 02447, Korea; bawoojang@khu.ac.kr (S.H.J.); 2017315113@khu.ac.kr (M.S.G.); tpfk3089@khu.ac.kr (Y.-J.J.); clearly1627@naver.com (M.-J.J.); waqser11@naver.com (J.S.C.); lya7575@gmail.com (Y.L.); 3 Kyung Hee East-West Pharmaceutical Research Institute, Kyung Hee University, Seoul 02447, Korea

**Keywords:** hesperidin, Alzheimer’s disease, neurogenesis, neural stem cells

## Abstract

Alzheimer’s disease (AD) is an irreversible neurodegenerative disease characterized by memory and cognitive impairments. Neurogenesis, which is related to memory and cognitive function, is reduced in the brains of patients with AD. Therefore, enhancing neurogenesis is a potential therapeutic strategy for neurodegenerative diseases, including AD. Hesperidin (HSP), a bioflavonoid found primarily in citrus plants, has anti-inflammatory, antioxidant, and neuroprotective effects. The objective of this study was to determine the effects of HSP on neurogenesis in neural stem cells (NSCs) isolated from the brain of mouse embryos and five familial AD (5xFAD) mice. In NSCs, HSP significantly increased the proliferation of NSCs by activating adenosine monophosphate (AMP)-activated protein kinase (AMPK)/cAMP-response element-binding protein (CREB) signaling, but did not affect NSC differentiation into neurons and astrocytes. HSP administration restored neurogenesis in the hippocampus of 5xFAD mice via AMPK/brain-derived neurotrophic factor/tropomyosin receptor kinase B/CREB signaling, thereby decreasing amyloid-beta accumulation and ameliorating memory dysfunction. Collectively, these preclinical findings suggest that HSP is a promising candidate for the prevention and treatment of AD.

## 1. Introduction

Alzheimer’s disease (AD) is a progressive neurodegenerative disease that causes impairment of memory, language function, spatial perception, and personality disorders [1]. The exact cause of AD is unclear. The brains of patients with AD are histologically characterized by the deposition of amyloid-beta (Aβ), hyperphosphorylated tau protein, and chronic inflammation [2,3,4]. In addition, reduced neurogenesis and neuronal loss occur in the brains of AD patients and animal models of AD [5,6]. Neurogenesis in the hippocampus continues throughout life. The hippocampus is involved in learning and cognitive abilities from embryonic development to adulthood [7,8,9]. Shors et al. demonstrated that decreased cellular proliferation in the hippocampus impairs learning and memory during trace conditioning [10]. Genetic deficiency of nestin, a neural stem cell marker, worsens memory loss in AD transgenic mouse models [11]. In contrast, increased neurogenesis, induced by the overexpression of cell cycle factors, enhances allocentric navigation, navigational learning, and contextual memory in mice [12]. Thus, many experiments have attempted to develop novel agents that can induce neurogenesis to treat neuronal-loss-related brain diseases.

Hesperidin (HSP) is a bioflavonoid found in plants belonging to the Citrus genus, including Citrus aurantium, C. sinensis, and C. unshiu [13]. The pharmacological activities of HSP include anti-inflammatory, anti-apoptotic, neuroprotective, and synaptic plasticity-promoting effects [14,15,16]. Welbat et al. reported that HSP ameliorated oxidative stress and enhanced neuroprotective effect in a methotrexate (MTX)-treated rat model [17]. HSP also activates adenosine monophosphate (AMP)-activated protein kinase (AMPK), a crucial signal in neurogenesis [18,19]. HSP ameliorated memory loss by increasing neurogenesis in an MTX-treated rat model [20]. Therefore, we hypothesized that HSP can alleviate AD pathology by increasing neurogenesis in five familial AD (5xFAD) mice. We focused on the neurogenetic effects of HSP and its possible role in neural stem cells (NSCs) isolated from embryonic mouse brains and AD transgenic mouse models.

## 2. Materials and Methods

### 2.1. Materials

Hesperidin (HSP; H0049) was purchased from Tokyo Chemical Industry (Tokyo, Japan). Deoxyribonuclease-1 (DN25) and 5-Bromo-2′-deoxyuridine (BrdU; B5002) were purchased from Sigma-Aldrich (St. Louis, MO, USA). Insulin-Transferrin-Selenium-Sodium Pyruvate (ITS-A; 51300044), neurobasal medium (21103049), fetal bovine serum (FBS; 10082139), bFGF recombinant human protein (PHG0368), EGF recombinant human protein (PHG0311), N-2 Supplement (17502-048), B27 supplement (17504-044), and GlutaMAX (35050079) supplement were purchased from Gibco (Waltham, MA, USA). Trypsin-EDTA solution (LS-015-08) and Dulbecco’s modified Eagle’s medium/F-12 Nutrient Mixture Ham (DMEM/F-12; LM002-05) were purchased from Welgene (Geongsan, Republic of Korea). Penicillin/Streptomycin (30010) was purchased from Hyclone Laboratories, Inc. (Logan, UT, USA). Alexa Fluor secondary antibodies were purchased from Thermo Fisher Scientific (Waltham, MA, USA).

### 2.2. Isolation and Culture of Neural Stem Cells

NSCs were dissociated from the cortex and hippocampus of embryonic (E) day 15.5 fetuses. The cortex and hippocampus were immersed in cold Ca^2+^/Mg^2+^-free Hanks’ balanced salt solution. Single cell suspensions were prepared by mechanical dissociation in DMEM/F12 supplemented with 10% penicillin/streptomycin, 1% N2 supplement, EGF (20 ng/mL), bFGF (20 ng/mL), and 10% ITS-A [21,22]. Dissociated cell suspensions were filtered through 40 µm cell strainers, and single cells were seeded at a density of 2.0 × 10^7^ cells per well in 100 mm cell culture dishes. After 3 days, half of the conditioned medium was replaced with fresh culture media.

### 2.3. Measurement of Cytotoxicity

**Figure 1:**
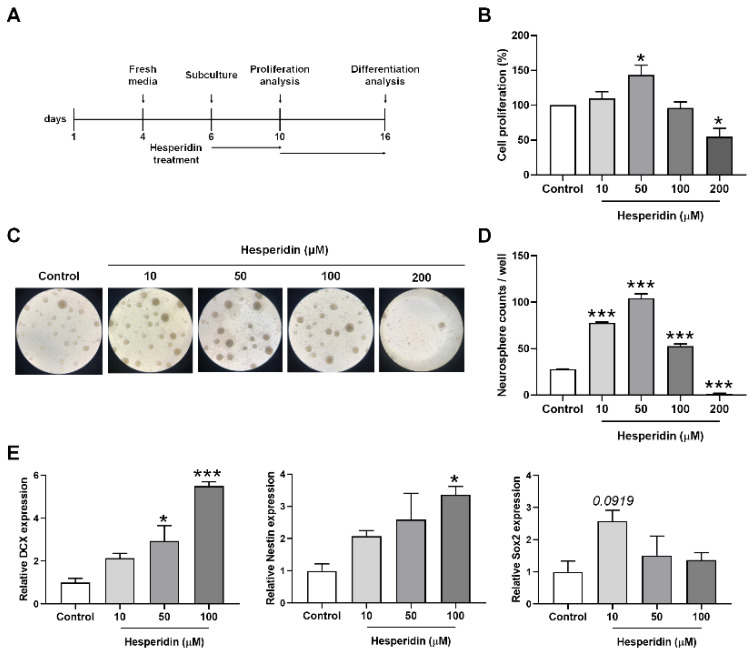
The effects of hesperidin on cell proliferation in neural stem cells isolated from mouse embryonic brain. (**A**) In vitro neurosphere culture and experiment scheme. Cells were pretreated with 10, 50, 100, and 200 µM hesperidin for 4 days. (**B**) Cell proliferation (%) was measured using a WST-1 assay. (**C**,**D**) Representative images showing proliferation and the number of neurospheres of at least 100 µm in the graph. (**E**) Neural stem cell-specific proliferation was confirmed by measuring the mRNA levels of DCX, nestin, and Sox2 by qRT-PCR. Statistical analysis included one-way analysis of variance and Tukey’s *post hoc* test. * *p* < 0.05 and *** *p* < 0.001 compared with the vehicle-treated control group. Data are shown as mean ± SEM (*n* = 3 per group).

Cell proliferation was measured using a water-soluble tetrazolium 1 (WST-1) assay. Second neurospheres were dissociated to single cells in suspension and seeded at a density of 1 × 10^4^ cells in 96-well plates. Cells were treated with HSP (0, 10, 50, 100, or 200 µM) for 48 h (Figure 1A). Conditioned media were replaced with serum-free 10% WST-1 solution. After 1 h, the absorbance of the media was measured using a microplate reader at 430 nm.

### 2.4. Neurosphere Counting

The single cells of secondary neurospheres were seeded into 96-well plates at a density of 1 × 10^4^ cells per well. Cells were treated with HSP (0, 10, 50, 100, or 200 µM). After 4 days, newly formed NSCs were counted using a Nikon Eclipse Ti-S/L100 microscope (Nikon; Tokyo, Japan). Neurospheres were defined by a minimum cutoff size of 100 µm.

### 2.5. Neural Stem Cells Differentiation Assay

Cells were mechanically dissociated in a neurobasal medium/DMEM/F12 medium supplemented with 10% presenilin/streptomycin, 1% B27 supplement, and 1% N2 supplement. For immunocytochemistry assays, secondary neurospheres were seeded on coverslips (12 mm) pre-coated with poly-L-lysine (10 µg/mL) in 24-well plates at a density of 1 × 10^3^ cells per well. For western blot assays, secondary neurospheres were plated on 6-well plates at a density of 1 × 10^6^ cells per well. Cells were analyzed on the eighth day after seeding.

### 2.6. Quantitative RT-PCR

RNA extraction and qRT-PCR were performed, as previously described [23]. RNA extraction was performed to use for Kit Hybrid-R™ (GeneAll; Seoul, Republic of Korea), and a Nanodrop ND-1000 spectrophotometer (Thermo Fisher Scientific) was used for measuring the concentration of RNA samples. About 3 or 5 µg of each RNA was converted to cDNA using TOPscript™ RT DryMIX (Enzynomics; Daejeon, Republic of Korea), and cDNA was quantified by the TOPscript™ RT DryMIX (Enzynomics). To confirm several transcriptions a mixture of the following components was prepared according to the indicated concentration: 3% forward and reverse primer, 4% SYBR Green PCR master mix. Primers were synthesized at Cosmo Genetech (Seoul, Republic of Korea). The following primers were used: Nestin (Gene ID: 18008; forward 5′-GAGGGAGAGGAAGAAGATGC-3′, reverse 5′-CACTGTCACCCACAGATTCA-3′), DCX (Gene ID: 13193; forward 5′-CTGGGGCTAGGGAAGTAGAG-3′, reverse 5′-ATTCTGAAGCTTCGGGTCTT-3′), Sox2 (Gene ID: 20674; forward 5′-ACTAGGGCTGGGAGAAAGAA-3′, reverse 5′-AGTGCAATTGGGATGAAAAA-3′), and GAPDH (Gene ID: 14433; forward 5′-TGAATACGGCTACAGCAACA-3′, reverse 5′-AGGCCCCTCCTGTTATTATG-3′).

### 2.7. Western Blot

Western blot analysis was performed, as previously described [24]. Initial cell lysis was used by RIPA buffer and protease/phosphatase inhibitors. Equal amounts of protein samples (25 or 50 µg) were separated to sodium dodecyl sulfate polyacrylamide gel and transferred to polyvinylidene difluoride membranes by electrophoresis. The membranes were washed with 0.1% Tween 20 in TBS buffer (TBST) and were reactivated in blocking solution (5% bovine serum albumin (BSA)). The membranes were then incubated with anti-AMPK (Cell signaling; Danvers, MA, USA, 2532), phospho-AMPK (Cell signaling; 2535), brain-derived neurotrophic factor (BDNF, Abcam; Cambridge, UK, ab108319), tropomyosin receptor kinase B (TrkB, Cell signaling; 4603), phospho-TrkA (Tyr785)/TrkB (Tyr816) (Cell signaling; 4168), cAMP response element-binding protein (CREB, Cell signaling; 9197), phospho-CREB (Ser133) (Cell signaling; 9198), or β-actin (Santa Cruz Biotechnology; Dallas, TX, USA, sc-47778HRP) overnight at 4 °C. All primary antibodies except β-actin (1:5000 dilution) were used at a 1:1000 dilution. After repeated washing times with TBST, the membranes were reacted with HRP-conjugated secondary antibodies (1:5000 dilution). Protein detection was performed using an ECL reagent (Bio-Rad Laboratories, Hercules, CA, USA) and captured by Solo6S EDGE (Vilber Lourmat Sté, Collégien, France).

### 2.8. Animals and Hesperidin Treatment

The 5xFAD mice were purchased from Jackson Laboratory (Bar Harbor, ME, USA; stock number: 034840-JAX). The mice (7-month-old) were housed at 23 ± 1 °C and 50 ± 10% humidity under a 12 h light/12 h dark cycle, with free access to food and water. HSP (100 mg/kg) was given orally once daily for two months. Mice were randomly divided into treatment groups; wild type (WT)+vehicle, WT+HSP, 5xFAD+vehicle, and 5xFAD+HSP. A month after the HSP treatment, BrdU (50 mg/kg) was injected intraperitoneally for five consecutive days. All animal studies were performed in accordance with the “*Guide for the Care and Use of Laboratory Animals*”, 8th edition, of the National Institutes of Health (2011), and approved by the “Animal Care and Use Guidelines” of Kyung Hee University (approval number: KHUASP(SE)-20-592).

### 2.9. Immunofluorescence

For BrdU/neuronal nuclei (NeuN) quantification in the hippocampus, we used 25 μm sections throughout the dentate gyrus at the start position of the hippocampus region. To ensure appropriate comparisons, we analyzed 3 sections from the similar bregma sections of each mouse. Sections were denatured to expose the antigen using 2N HCl for 1 h at 37 °C and neutralized by washing three times with boric acid. After rinsing three times with PBS, the sections were blocked for 1 h in PBS containing 3% normal goat serum, 1% BSA, and 0.4% Triton X-100. The sections were then washed three times with PBS and incubated overnight in anti-BrdU (1:100 dilution) or anti-NeuN (1:300 dilution) antibodies at 4 °C. After repeated washing times, the sections were incubated with Alexa Fluor-488 or 594-conjugated secondary antibodies (1:1000 dilution) at room temperature. Finally, each section was mounted on slide glasses. The immunolabeled sections were observed with a K1-Fluo confocal microscope (Nanoscope Systems, Daejeon, Republic of Korea).

### 2.10. Thioflavin S Staining

The sections were washed with PBS and mounted on gelatin-coated glass slides. The slides were incubated at a 0.5% Thioflavin S (ThS) for 5 min. Next, we washed the slides two times: with 50% ethanol and distilled water for 5 min each, and then covered the slides with mounting medium.

### 2.11. Morris Water Maze Test

The Morris water maze (MWM) was performed, as previously described [25]. Mice were adapted to the maze one day before training. A platform was assigned to each mouse and set at a fixed position throughout the training session. Mice were placed at different starting points in a room composed of cues on different sides. The training session was composed of a series of three trials per day for seven days. The time to mount the platform was recorded and the average value was used. On day eight, a probe test was performed without the platform for 60 s.

### 2.12. Statistical Analysis

All data are presented as the mean ± standard error of the mean. The differences between the groups were analyzed with the Students’ *t* tests. One-way or two-way ANOVA followed by Tukey’s multiple comparisons test was used for multiple group comparisons. Statistical analyses were performed using Graph Pad Prism 8.0 software (Graph Pad Software Inc., San Diego, CA, USA). A value of *p* < 0.05 was considered a significant difference.

## 3. Results

### 3.1. HSP Increased Cell Proliferation

To measure the effect of HSP on cell proliferation, we performed a WST-1 assay. The proliferation of NSCs was significantly increased following treatment with 50 μM of HSP for 24 h, compared with proliferation in vehicle-treated NSCs (Figure 1B). As NSCs form neurospheres that proliferate undifferentiated neurons, we investigated the changes in neurosphere counts following HSP treatment. HSP treatment dramatically increased the number of neurospheres compared with the number of neurospheres in vehicle-treated NSCs (Figure 1C,D). The mRNA levels of the cell proliferation markers (DCX, nestin, and Sox2) were upregulated in HSP-treated NSCs in a dose-dependent manner compared to the mRNA levels in vehicle-treated NSCs (Figure 1E).

Next, to determine whether HSP also affects cell differentiation, we analyzed cell-type differentiation in the neurospheres. Differentiated cells were stained with the β3-tubulin and glial fibrillary acidic protein (GFAP) antibodies, which are used to identify neurons and astrocytes, respectively [26]. No significant changes in β3-tubulin^+^ and GFAP^+^ cells were detected after HSP treatment compared with vehicle-treated cells (Figure S1A,B). Western blotting showed similar results (Figure S1C,D). These results indicate that HSP affects cell proliferation rather than cell differentiation.

### 3.2. HSP Activated AMPK/CREB Signaling in Neural Stem Cells

**Figure 2:**
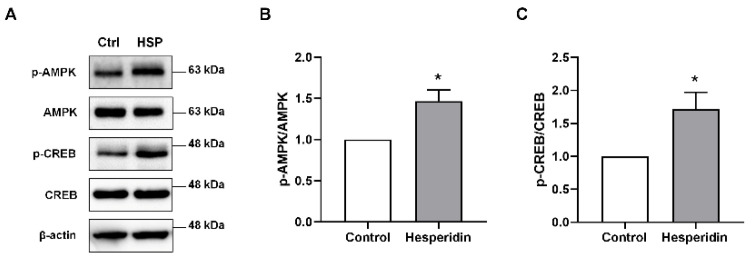
The effects of hesperidin on AMPK/CREB signaling in neurospheres. To prove the neurogenic effects of HSP, we measured AMPK and CREB protein levels in vitro. Phosphorylated/total AMPK and CREB were analyzed by western blot. (**A**) Representative images of AMPK and CREB in neurosphere lysates. (**B**,**C**) Quantification of AMPK (**B**) and CREB (**C**) expression. Statistical analysis included Student’s *t* test. * *p* < 0.05 compared with the vehicle-treated control group. Data are shown as mean ± SEM (*n* = 3 per group).

AMPK/CREB signaling contributes to NSC proliferation [27]. Thus, we investigated whether the increase in cell proliferation following HSP treatment was related to AMPK/CREB signaling. The protein levels of phosphorylated AMPK and CREB increased significantly in HSP-treated NSCs compared with the protein levels in vehicle-treated NSCs (Figure 2). These data suggest that HSP increases cell proliferation by activating AMPK/CREB signaling in NSCs.

### 3.3. HSP Increased Hippocampal Neurogenesis in 5xFAD Mice

**Figure 3:**
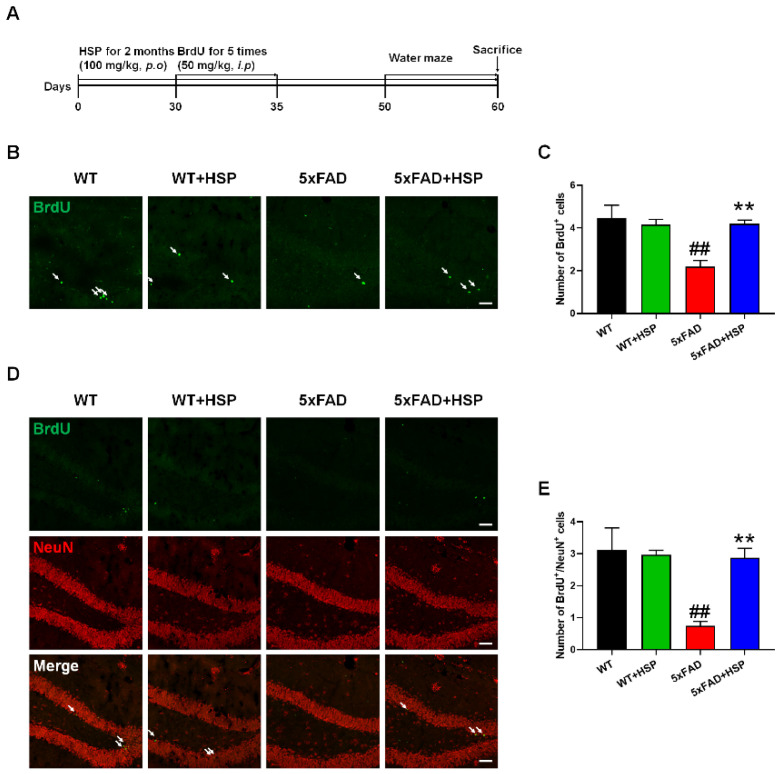
The effects of hesperidin on neurogenesis in the hippocampus of 5xFAD mice. Mice were administered with vehicle or HSP (100 mg/kg) once daily for two months. (**A**) In vivo experiment scheme. (**B**) Representative images of BrdU^+^ cells in the hippocampus. (**C**) The quantification of BrdU^+^ cells. (**D**) Representative images of BrdU^+^(green)NeuN^+^(red) cells in the hippocampus. (**E**) The quantification of BrdU^+^(green)NeuN^+^(red) cells. Scale bar; 50 μm. Arrows; BrdU^+^ cells (**B**) or BrdU^+^NeuN^+^ cells (**D**). Statistical analysis included one-way analysis of variance and Tukey’s *post hoc* test. ^##^ *p* < 0.01 compared with the vehicle-treated WT mice. ** *p* < 0.01 compared with the vehicle-treated 5xFAD mice. Data are shown as mean ± SEM (*n* = 4 per group).

To confirm the proliferative effects of HSP in vivo, we orally administered HSP to 7-month-old 5xFAD mice for two months (Figure 3A). First, brain sections were stained with a BrdU-specific antibody to analyze cell proliferation in the subgranular zone (SGZ) of the hippocampus. BrdU-positive cells in the SGZ were significantly lower in the 5xFAD mice compared to that of WT mice. However, the number of BrdU-positive cells was significantly higher in the brains of HSP-treated 5xFAD mice compared with that of vehicle-treated 5xFAD mice (Figure 3B,C). Additionally, to determine whether the HSP-stimulated BrdU^+^ cells differentiated into mature neurons, we performed double-staining using anti-BrdU and NeuN (a mature neuronal cell marker). As expected, the number of BrdU^+^NeuN^+^ cells was lower in the hippocampus of 5xFAD mice compared to that in WT mice. Contrarily, HSP-administered 5xFAD mice showed dramatically increased BrdU^+^NeuN^+^ cell counts compared with that of the vehicle-treated 5xFAD mice (Figure 3D,E). These results demonstrate that HSP exerted cell proliferative effects and increased the number of newborn neurons in 5xFAD mice.

### 3.4. HSP Activated AMPK/BDNF/TrkB/CREB Signaling in the Hippocampus of 5xFAD Mice

**Figure 4:**
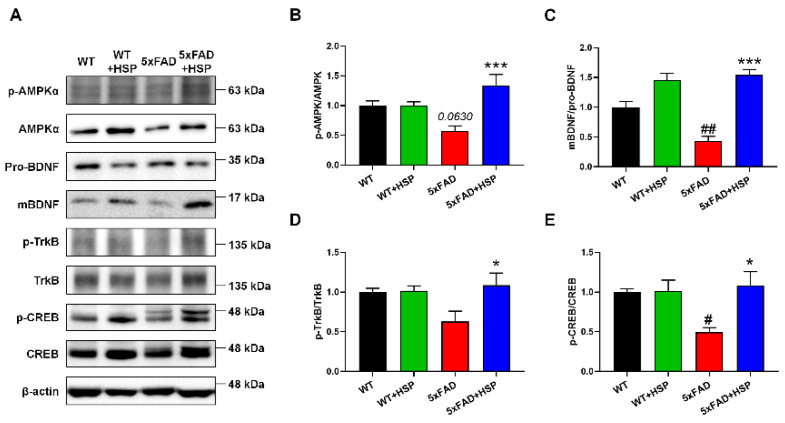
The effects of hesperidin on AMPK/BDNF/TrkB/CREB signaling in the hippocampus of 5xFAD mice. (**A**–**E**) Representative images of western blots (**A**) and quantification of AMPK (**B**), BDNF (**C**), TrkB (**D**), and CREB (**E**). Statistical analysis included one-way analysis of variance and Tukey’s *post hoc* test. ^#^ *p* < 0.05 and ^##^ *p* < 0.01 compared with the vehicle-treated WT mice. * *p* < 0.05 and *** *p* < 0.001 compared with the vehicle-treated 5xFAD mice. Data are shown as mean ± SEM (*n* = 4 per group).

Western blotting was performed to identify the mechanism by which HSP enhances neurogenesis in 5xFAD mice (Figure 4A). HSP is an AMPK activator involved in neurogenesis [27]. Thus, we measured the protein expression of phosphorylated AMPK in the hippocampi of mice. Phosphorylated AMPK levels were slightly decreased in 5xFAD mice compared to that in WT mice; however, this protein was significantly upregulated by HSP treatment in the 5xFAD mice. (Figure 4B). Next, we analyzed the protein levels of BDNF and TrkB, which are important for neurogenesis, in the hippocampi of the mice [28]. HSP treatment increased the levels of mature BDNF and phosphorylated TrkB in the hippocampi of 5xFAD mice (Figure 4C,D). We also detected the levels of phosphorylated CREB activated by BDNF/TrkB signaling. In 5xFAD mice, the protein levels of phosphorylated CREB were markedly lower than that in WT mice. However, HSP-treated 5xFAD mice exhibited dramatically higher levels of this protein compared with the levels in vehicle-treated 5xFAD mice (Figure 4E). These findings suggest that HSP increased hippocampal neurogenesis by mediating AMPK/BDNF/TrkB/CREB signaling.

### 3.5. HSP Ameliorated Memory Impairment and Aβ Accumulation in 5xFAD Mice

**Figure 5:**
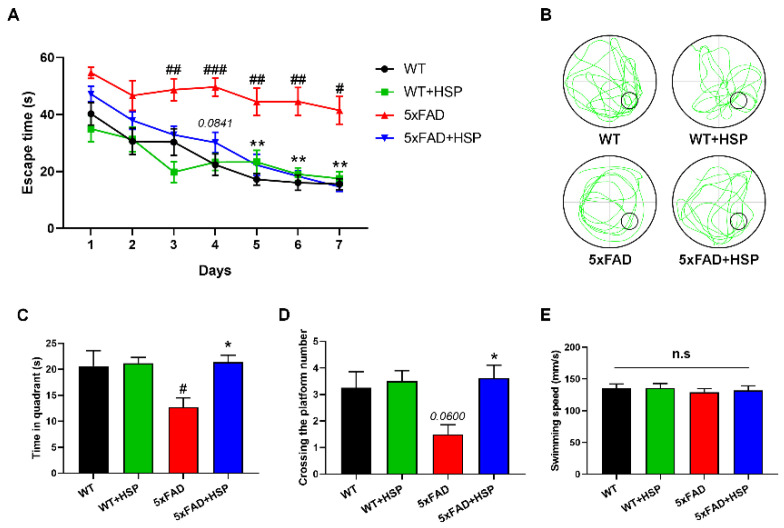
The effects of hesperidin on spatial memory functions in 5xFAD mice. (**A**) Escape time (s) of mice over 7 days. (**B**) Representative swimming paths on day 8. (**C**–**E**) On day 8 of the probe trial, time in quadrant (**C**), crossing the platform number (**D**), and swimming speed (**E**) were recorded. Statistical analysis included one- or two-way analysis of variance and Tukey’s *post hoc* test. ^#^ *p* < 0.05, ^##^ *p* < 0.01, and ^###^ *p* < 0.001 compared with the vehicle-treated WT mice. * *p* < 0.05 and ** *p* < 0.01 compared with the vehicle-treated 5xFAD mice. Data are shown as mean ± SEM (WT mice, *n* = 12; WT+HSP mice, *n* = 12; 5xFAD mice, *n* = 12; 5xFAD+HSP mice, *n* = 13). n.s; not significant.

Increased neurogenesis can affect learning and memory function [12]. Therefore, 5xFAD mice were subjected to MWM testing to determine the effects of HSP-induced neurogenesis on memory function. The time taken to reach a hidden platform within the water tank was measured. All groups reached the platform at similar times on the first day of training; however, the 5xFAD mice had significantly longer escape times compared with WT mice from day three onwards. HSP-treated 5xFAD mice showed markedly faster escape times than vehicle-treated 5xFAD mice from day five onwards (Figure 5A). Additionally, the platform on the eighth day was removed, and a probe test was performed. HSP administration increased the time spent in the quadrant and the number of crossings of the platform without decreasing locomotor activity in 5xFAD mice (Figure 5B–E). These results indicated that the effect of HSP on hippocampal neurogenesis improved cognitive impairment in 5xFAD mice. As accumulated Aβ is a major hallmark of AD, we performed ThS staining to detect Aβ plaques. HSP reduced the ThS-positive areas (%) in the brains of the 5xFAD mice (Figure S2).

## 4. Discussion

In this study, we demonstrated the neurogenic effects of HSP in NSCs and 5xFAD mice. NSCs isolated from mouse embryonic brains were treated with HSP to investigate the cell proliferative effects of HSP. HSP increased cell proliferation (%), neurosphere counts, and the mRNA levels of cell proliferation factors (DCX, nestin, and Sox2), but did not affect cell differentiation. HSP also activated AMPK/CREB signaling in NSCs. We proved that HSP promotes hippocampal neurogenesis by increasing AMPK/BDNF/TrkB/CREB signaling, resulting in the alleviation of memory dysfunction in 5xFAD mice.

Neurogenesis is the formation of new neurons in the brain. It is a critical process during fetal development, and also persists in particular brain regions (the SVZ and SGZ) throughout the human lifespan [29]. Neurogenesis greatly influences memory and cognition [30]. Reduced neurogenesis has been observed in the brains of AD patients and mouse models of AD [5,6]. Reduced hippocampal neurogenesis induced by nestin deficiency, a cell proliferation marker, aggravates memory and cognitive deficits in APP/PS1 mice [11]. Several studies have demonstrated that enhanced neurogenesis improves memory impairment in AD mouse models. For instance, in APP/PS1 mice, andrographolide ameliorated spatial memory dysfunction by promoting cell proliferation without altering neuronal differentiation [31]. Valproic acid increased NSC proliferation in the SGZ of the hippocampus, resulting in improved cognitive abilities in APP/PS1 mice [32]. These findings indicate that promoting neurogenesis may ameliorate cognitive deficits. Our findings also demonstrate that HSP-stimulated hippocampal neurogenesis attenuates the memory impairment of 5xFAD mice.

Although the mechanisms involved in neurogenesis are not fully known, BDNF and CREB are essential signals in this process. In the central nervous system, BDNF and its high-affinity receptor TrkB play critical roles in neuronal survival and function [33]. Injections of BDNF into the hippocampus of adult rats increased the number of newly generated neurons [34], and BDNF knockdown in rat hippocampi decreased neurogenesis [35]. BDNF/TrkB signaling leads to phosphorylation of CREB, which is involved in memory formation [36]. Phosphorylated CREB decreased in the brains of patients and mice with AD [37,38]. Hong et al. demonstrated that the activation of BDNF/TrkB/CREB signaling ameliorates cognitive deficits in APP/PS1 mice, indicating that BDNF and CREB influence learning and memory through neurotrophic and memory consolidation effects. Moreover, the expression of BDNF and CREB is enhanced by AMPK activation, which leads to increased neurogenesis [39,40]. HSP, a flavanone glycoside found in citrus fruits, activates AMPK [41]. In this study, we found that HSP promoted AMPK phosphorylation in NSCs and the hippocampus, suggesting that HSP can increase neurogenesis via AMPK activation.

In conclusion, this study revealed the neurogenic effects of HSP in NSCs and 5xFAD mice. We found that HSP increased NSC proliferation and the number of mature neuronal cells, thereby ameliorating cognitive impairment in 5xFAD mice. Furthermore, the cell proliferative effects of HSP were mediated by the activation of the AMPK/BDNF/CREB signaling pathway. Collectively, these findings suggest that HSP is a neurogenesis enhancer and may be a potential candidate for AD treatment.

## Supplementary Materials

The following supporting information can be downloaded at: https://www.mdpi.com/article/10.3390/nu14153125/s1, Figure S1: The effects of hesperidin on cell differentiation in neural stem cells isolated from mouse embryonic brain. (**A**) Immunofluorescence images showing β3-tubulin+ and GFAP^+^ cells. (**B**) Quantification of β3-tubulin^+^ and GFAP^+^ cells (%). (**C**,**D**) Representative images of western blots (**C**) and quantification of β3-tubulin and GFAP (**D**). Statistical analysis included Student’s *t* test. Data are shown as mean ± SEM (*n* = 3 per group). n.s; not significant; Figure S2: The effect of hesperidin on Aβ accumulation in the brain of 5xFAD mice. (**A**) Representative images of ThS^+^ areas in the hippocampus. (**B**) The quantification of ThS^+^ areas (%). Scale bar; 50 μm. Statistical analysis included Student’s *t* test. ** *p* < 0.01 compared with the vehicle-treated 5xFAD mice. Data are shown as mean ± SEM (*n* = 5 per group). HSP; hesperidin.
