# Supplementary material for: Hesperidin Improves Memory Function by Enhancing Neurogenesis in a Mouse Model of Alzheimer’s Disease
Source: Nutrients. 2022 Jul 29;14(15):3125. doi: 10.3390/nu14153125 (PMC9370591; doi:10.3390/nu14153125)
Supplement: Supplementary file 1 [file nutrients-14-03125-s001.zip › nutrients-1827872-supplementary.pdf]

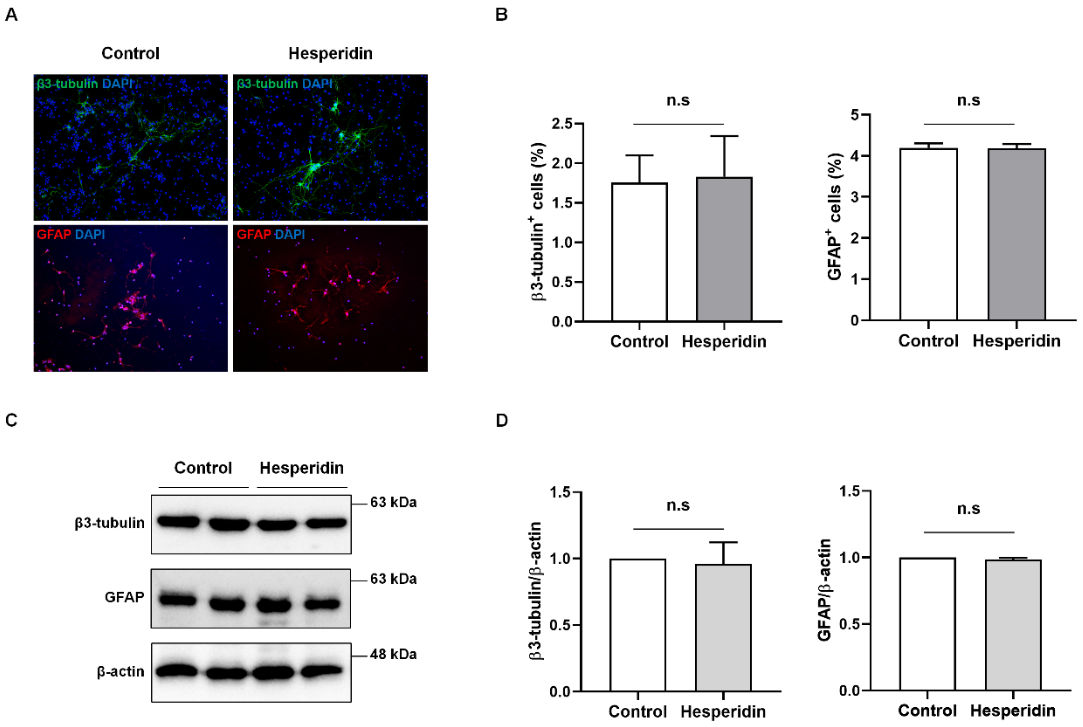

Supplementary Figure S1. The effects of hesperidin on cell differentiation in neural stem cells isolated from mouse embryonic brain. (**A**) Immunofluorescence images showing β3-tubulin$^+$ and GFAP$^+$ cells. (**B**) Quantification of β3-tubulin$^+$ and GFAP$^+$ cells (%). (**C**,**D**) Representative images of western blots (**C**) and quantification of β3-tubulin and GFAP (**D**). Statistical analysis included Student's *t* test. Data are shown as mean ± SEM (n = 3 per group). n.s; not significant.

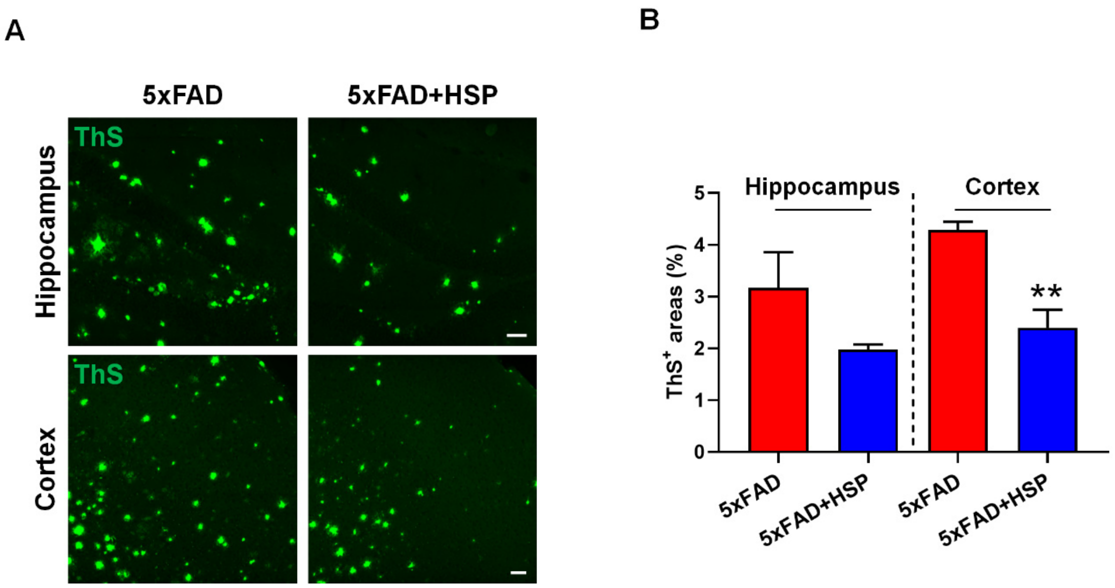

Supplementary Figure S2. The effect of hesperidin on Aβ accumulation in the brain of 5xFAD mice. (**A**) Representative images of ThS$^+$ areas in the hippocampus. (**B**) The quantification of ThS$^+$ areas (%). Scale bar; 50 μm. Statistical analysis included Student's *t* test. ** $p < 0.01$ compared with the vehicle-treated 5xFAD mice. Data are shown as mean ± SEM (n = 5 per group). HSP; hesperidin.
